# Indian consensus on chronic constipation in adults: A joint position statement of the Indian Motility and Functional Diseases Association and the Indian Society of Gastroenterology

**DOI:** 10.1007/s12664-018-0894-1

**Published:** 2019-01-08

**Authors:** Uday C. Ghoshal, Sanjeev Sachdeva, Nitesh Pratap, Abhai Verma, Arun Karyampudi, Asha Misra, Philip Abraham, Shobna J. Bhatia, Naresh Bhat, Abhijit Chandra, Karmabir Chakravartty, Sujit Chaudhuri, T. S. Chandrasekar, Ashok Gupta, Mahesh Goenka, Omesh Goyal, Govind Makharia, V. G. Mohan Prasad, N. K. Anupama, Maneesh Paliwal, Balakrishnan S. Ramakrishna, D. N. Reddy, Gautam Ray, Akash Shukla, Rajesh Sainani, Shine Sadasivan, Shivaram P. Singh, Rajesh Upadhyay, Jayanthi Venkataraman

**Affiliations:** 1 Department of Gastroenterology, Sanjay Gandhi Postgraduate Institute of Medical Sciences, Lucknow, 226 014 India; 2 G B Pant Hospital, Jawaharlal Nehru Marg, Delhi, 110 002 India; 3 KIMS Hospital, Secunderabad, 500 003 India; 4 Asian Institute of Gastroenterology, Hyderabad, 500 082 India; 5 P D Hinduja Hospital and MRC, and Hinduja Heathcare Surgical, Mumbai, 400 016 India; 6 KEM Hospital, Mumbai, 400 012 India; 7 Aster CMI Hospital, Bangalore, 560 092 India; 8 King George Medical University, Lucknow, 226 003 India; 9 Poddar Institute of Gastrointestinal and Liver Disease, Kolkata, 700 053 India; 10 Advanced Medicare Research Institute, Salt Lake, Kolkata, 700 091 India; 11 Department of Gastroenterology, Medindia Hospitals, Nungambakkam, Chennai, 600 034 India; 12 Department of Surgical Gastroenterology, Sanjay Gandhi Postgraduate Institute of Medical Sciences, Lucknow, 226 014 India; 13 Appollo Gleneagles Hospitals, Kolkata, 700 054 India; 14 Dayanand Medical College, Ludhiana, 141 001 India; 15 All India Institute of Medical Sciences, New Delhi, 110 029 India; 16 VGM Hospital, Coimbatore, 641 005 India; 17 Yatharth Super Speciality Hospitals, Noida, 201 304 India; 18 SRM Institutes for Medical Science, Chennai, 600 026 India; 19 B R Singh Railway Hospital, Kolkata, 700 014 India; 20 Lokmanya Tilak Municipal General Hospital and Medical College, Sion, Mumbai, 400 022 India; 21 Jaslok Hospital, Mumbai, 400 026 India; 22 Amrita Institute of Medical Science, Cochin, 682 041 India; 23 S.C.B. Medical College, Manglabag, Cuttack, 753 007 India; 24 Max Superspeciality Hospital, New Delhi, 110 088 India; 25 Gleneagles Global Health City, Chennai, 600 100 India

**Keywords:** Bristol stool form, Colon transit, Fecal evacuation disorder, Functional gastrointestinal disorders, Irritable bowel syndrome

## Abstract

The Indian Motility and Functional Diseases Association and the Indian Society of Gastroenterology developed this evidence-based practice guideline for management of chronic constipation. A modified Delphi process was used to develop this consensus containing 29 statements, which were generated by electronic voting iteration as well as face to face meeting and review of the supporting literature primarily from India. These statements include 9 on epidemiology, clinical presentation, and diagnostic criteria; 8 on pathophysiology; and the remaining 12 on investigations and treatment. When the proportion of those who voted either to accept completely or with minor reservation was 80% or higher, the statement was regarded as accepted. The members of the consensus team believe that this would be useful for teaching, clinical practice, and research on chronic constipation in India and in other countries with similar spectrum of the disorders.

## Introduction

The Indian Motility and Functional Diseases Association was formed at the Department of Gastroenterology, Sanjay Gandhi Postgraduate Institute of Medical Sciences, Lucknow, in May 2011 with the following aims: (a) to help physicians to provide a standardized care to patients suffering from gastrointestinal motility and functional disorders; (b) to share knowledge about these diseases among doctors and other health care workers; (c) to increase awareness about these diseases with patients, public, and policy maker; and (d) to perform multicentric research on these diseases. The association was registered in Lucknow, India, on May 21, 2011.

Since its inception, the association organized several educational congresses to spread awareness about various functional gastrointestinal and motility disorders including a major international congress of the Asian Neurogastroenterology and Motility Association in New Delhi, India, on February 6–8, 2015. However, it was realized that there is a need to undertake scientific activities on issues somewhat unique to India. Hence, the current consensus activity, which is the first such activity by the Association, was undertaken; the Indian Society of Gastroenterology, the major Gastroenterology Society of the country, also joined hands to undertake this consensus.

Chronic constipation (CC) is not an uncommon problem in Indian communities and in clinical practice [1]. The prevailing belief of the Indian experts for several years that the epidemiology, clinical spectrum, diagnostic assessment, treatment need, and patient expectations among patients with CC in India were somewhat different compared to the West have been recently reviewed in an article that antedated this consensus publication [1]. Therefore, the importance of reviewing the published Indian data was felt necessary, and in absence of adequate literature, a decision based on the collective experience of experts from India had to be put forward to guide clinicians managing these patients. Accordingly, the present consensus on CC was undertaken.

## Methods

The members of the consensus team were selected among Indian gastroenterologists based on their interest on CC as evidenced by an electronic literature search, their clinical interest, and recommendation of the sponsoring societies. A core group was selected from the consensus team members, who made the first set of 35 statements on epidemiology, clinical presentation, diagnostic criteria, pathophysiology, investigation, and treatment. The consensus process involved a modified Delphi method described previously [2]. Before the first voting on the statements, an electronic library was made in the Digital Medical Education section of the Shanti Public Educational and Development Society website (www.spreadhealth.in). Subsequently, each statement was discussed in a face to face meeting and voted during the 4th Biennial Conference of the Indian Motility and Functional Diseases Association at Hyderabad on April 30, 2017. Based on the results of the initial voting, the statements were modified and revised resulting in deletion of 6 statements. At this stage, a review article on Indian data on CC was written by one of the members [1] and published in the *Indian Journal of Gastroenterology* and made available to the other members. The revised statements were voted again in an electronic online anonymous voting system developed in the Research and Innovation initiative menu in the www.spreadhealth.in and the results analyzed electronically on October 12, 2017. The grade of the evidence and the level of agreement were based on the method of the Grading of Recommendations Assessment, Development and Evaluation (GRADE) Working Group (see Table 1) [3]. When the proportion of those who voted either to accept completely or with minor reservation was 80% or higher, the statement was regarded as accepted. However, an occasional important statement was retained even when it did not receive the needed voting to stimulate further research on this issue. Finally, consensus was achieved on the following 29 statements. Subsequently, the results of the consensus were presented to the members of the Indian Motility and Functional Diseases Association on November 11, 2017 at a mid-term meeting of the association endorsed by the Rome Foundation at Lucknow, India, and to the members of the Indian Society of Gastroenterology at its 58th Annual Conference at Bhubaneswar, India, on December 17, 2017.

**Table 1:** Level of the agreement, level of evidence, and recommendation used in this consensus

Level of agreement	
I	Accepted completely
II	Accepted with some reservation
III	Accepted with major reservation
IV	Rejected with reservation
V	Rejected completely
Level of evidence	
I	Evidence obtained from at least one randomized controlled trial
II-1	Evidence obtained from well-designed controlled trials without randomization
II-2	Evidence obtained from well-designed cohort or case-controlled study
II-3	Evidence obtained from the comparison between time and places with or without intervention
III	The opinion of respected authorities, based on experience or expert committees
Recommendation (based on the quality of evidence)	
A	There is good evidence to support the statement
B	There is fair evidence to support the statement
C	There is poor evidence to support the statement but recommendation made on other grounds
D	There is fair evidence to refute the statement
E	There is good evidence to refute the statement

## Consensus statements

### Epidemiology, clinical presentation, and diagnostic criteria

#### Statement No. 1. Chronic constipation is common in India

Voting summary: Accepted completely: 23 (79%), accepted with some reservation: 3 (10%), accepted with major reservation: 3 (10%)

Level of evidence: II-2

Grade of recommendation: B

In spite of the scarcity of data, available studies indicate CC to be a common health problem in India, contradicting the popular belief of its infrequency due to vegetarianism with high fiber intake, and higher frequency of bowel movement, suggesting that CC might be, in fact, under-reported (Table 2) [1]. In a northern Indian community study, 555/4767 (11.6%) reported symptoms of constipation [4]. In a larger pan-Indian multicentric study, 2785 patients with chronic lower gastrointestinal symptoms with no organic cause and 4500 non-complaining subjects were interviewed. In the former group, 1404 (53%) had self-perceived constipation, while in the latter, 846 (18%), 1030 (23%) reported straining at stools, and incomplete stool evacuation, respectively [5]. In another community survey in rural northern India, 698/2774 (25%) passed predominantly Bristol types I–III stools and the prevalence of constipation-predominant irritable bowel syndrome (IBS-C) was 2.4% [6]. Two smaller community surveys from Chandigarh and Bangalore reported the prevalence of constipation of 24.8% and 8.6%, respectively, though the latter study was conducted exclusively among elderly population [7, 8]. A meta-analysis of 45 community studies from outside India suggested a global prevalence of CC to be 14% [9].

**Table 2:** Community studies on chronic constipation in India

Author	Place	Sample size	Criteria for diagnosis	Prevalence of constipation (%)
Makharia et al. 2011 [4]	Rural Haryana	4767	Self-perception	11.6
			Rome III (for IBS-C)	0.3
Ghoshal et al. 2008 [5]	Multicentric (22 centers)	Complainants: 2785	Self-perception	53
		Non-complainants: 4500	Self-perception	~ 41
Ghoshal and Singh 2017 [6]	Rural Uttar Pradesh	2774	Rome III	2.4
Rajput and Saini 2014 [7]	Chandigarh	505	Rome II	16.8
			Self-perception	24.8
Panigrahi et al. 2013 [14]	Odisha	1200	≤ 3 stools/week	2.6

*IBC-C* constipation-predominant irritable bowel syndrome

The female population is expected to suffer from CC more frequently than male due to slower transit, pelvic floor dysfunction due to obstetric trauma, harder stool forms, and over-reporting [10–13]. In a coastal eastern Indian study on 1200 subjects, female population had lesser frequency (11.1 ± 5.7 vs. 12.8 ± 3.8 stools per week; *p* < 0.001) and harder forms of stools (Bristol I, 17 (3.5%) vs. 6 (0.8%); Bristol II, 20 (4.1%) vs. 18 (2.5%); Bristol III, 39 (8%) vs. 60 (8.4%); *p* < 0.001) compared to males. Female population had a greater reduction of stool frequency with increasing age compared to males [14]. In the earlier community study from Chandigarh on 505 subjects, CC (Rome II criteria), present in 16.8%, was more common in females than males (20% vs. 12%, *p* = 0.041) [7]. More studies are needed on this issue.

The frequency of CC increases with age [14]. In the coastal eastern Indian study, stool frequency reduced with age, particularly among female [14]. In another study, of 925 patients with constipation, patients with functional constipation (FC) were older than those with IBS [15]. In an eastern Indian study, of 331 consecutive patients with CC, 65% were older than 60 years [1, 16, 17].

#### Statement No. 2. Stool frequency is higher and forms softer in India

Voting summary: accepted completely 21 (72%), accepted with some reservation 7 (28%)

Level of evidence: II-2

Grade of recommendation: B

Colon transit time is known to correlate inversely with Bristol stool forms; shorter transit time is associated with higher scores [18]. Accordingly, the Indian patients have softer stools than the patients from the West, for example the USA [19]. Stool weight is also high in India. In an initial community study [20], stool weight and whole-gut transit time among 550 healthy individuals were 311 g per 24 h and 39.8 h, respectively, which were considerably different from that reported from the West [20]. Stool frequency is also high in Indian population. In a pan-Indian multicentric study, the average daily stool frequencies in 4500 subjects were > 3, 3, 2, 1, and < 3/week in 3.7%, 5.4%, 34%, 56%, and 1%, respectively [5]. In a community study from rural northern Indian state (Uttar Pradesh), median stool frequency of 2774 subjects was 2/day (range 1 to 4). Bristol type IV, V, VI, and VII stools were passed by 39.5%, 26%, 7%, and 3% subjects, respectively [6]; the corresponding figures from the study conducted in coastal eastern Indian state were 50%, 6%, 18%, and 0.6%, respectively [14].

#### Statement No. 3. Constipation should be defined in India by stool forms and patients’ perception rather than by stool frequency

Voting summary: accepted completely 23 (79%), accepted with some reservation 4 (14%), accepted with major reservation 1 (3%), rejected with reservation 1 (3%)

Level of evidence: II-2

Grade of recommendation: A

As the daily stool frequency is higher in the Indian population, a component of the western definition of CC as a stool frequency < 3/week is expected to be insensitive and, hence, not applicable in the Indian setting to diagnose CC. In a pan-Indian multicentric study on 2656 patients with chronic lower GI symptoms, without alarm features and negative investigations, the median stool frequency (14 per week) was similar among patients who had self-perceived diarrhea or constipation [5]. Though 1404 patients reported experiencing constipation, only 507 (36%) of them had a stool frequency < 3 per week [5]. In another multicentric Indian irritable bowel syndrome (MIIBS) study, of 1618 patients with chronic lower GI symptoms, 462 (28.6%) had self-perceived  constipation [21]. Applying the stool frequency criteria, only 319 (19.7%) patients were diagnosed as CC. However, constipation was diagnosed in a greater proportion (655, 40.5%) of patients by the Bristol stool form criteria (stool types I–III) [21]. Patient perception is important for assessment of subjective symptoms and categorization of non-organic GI disorders that are diagnosed by symptom-based criteria. Some Indian data do suggest relationship between patient-perceived incomplete evacuation and straining and abnormal physiology among patients with CC [22]. Moreover, studies from India (MIIBS study) as well as the West suggest that patients' perception, which not only depends on pathophysiology but also on socio-cultural factors determine symptom reporting and may increase the diagnostic sensitivity of symptom-based criteria [21, 23, 24].

#### Statement No. 4. Constipation-associated stools, defined as Bristol types I–III, increase diagnostic sensitivity of CC in India than types I–II, as defined in the West

Voting summary: accepted completely 19 (65.5%), accepted with some reservation 5 (17%), rejected with reservation 4 (13.8%)

Level of evidence: II-2

Grade of recommendation: B

As Indian people pass softer stools, defining CC by stool forms as Bristol types I–II, as proposed in the West, might overlook a large proportion of patients. In the study conducted in the rural Uttar Pradesh, of 190 patients with IBS, 9 (4.7%) were classified as IBS-C using stool form criteria of Bristol types I–II; when Bristol types I–III was used to denote CC, the proportion increased to 33% [6]. In an eastern Indian study evaluating 331 patients with CC, though only 68% were classified as having constipation using Bristol stool types I–II, by adding type III, the proportion increased to 94% [16]. The results of a multicentric Indian IBS (MIIBS) study also supported the inclusion of type III stool to denote constipation in addition to types I and II in India [21].

#### Statement No. 5. As abdominal pain is less in frequency and severity, functional constipation is more common than IBS-C in India

Voting summary: accepted completely 17 (58.6%), accepted with some reservation 10 (34.5%), rejected completely 1 (3%)

Level of evidence: II-2

Grade of recommendation: B

Quite a few Indian studies reported FC to be more common than IBS-C. A prospective study on 925 patients with CC reported that 75.6% and 24.4% had FC and IBS-C, respectively, using the Rome III criteria [15]. Patients with FC were older than IBS-C in this study [15]. A prospective study from Kolkata, West Bengal, showed that FC was diagnosed in 69% and IBS-C in 13.8% of the 331 patients presenting with CC [16]. Similar observations have been made by two other Indian studies [25, 26] (Table 3). A higher proportion of FC than IBS-C in India is quite expected as abdominal pain, which is essential to diagnose IBS according to the Rome criteria, is less in frequency and severity among Indian patients with IBS [27].

**Table 3:** Studies comparing functional constipation and constipation-predominant irritable bowel syndrome among patients with chronic constipation using Rome III criteria

Author		Sample size	Frequency (%)	Mean age (years)	Sex (M/F)	Risk factors
Rooprai et al. 2017 [15]		925 CC				
	FC	699	75.6	46.8	1.7:1	Hypertension, diabetes mellitus
	IBS-C	226	24.4	43.8	1.9:1	Acid peptic disease
Ray 2016 [16]		331 CC				
	FC	224	69	63.4	1.1:1	Diabetes mellitus, hypothyroid, organic brain disease, drugs
	IBS-C	36	13.8	33.7	1.6:1	
Shah et al. 2014 [25]		99 CC				
	FC	74	75	53	–	
	IBS-C	25	25	55	–	
Ghoshal 2017 [1]		96 FED				
	FC	64	66		6.1:1	
	IBS-C	28	29		4.6:1	

*CC* chronic constipation, *FC* functional constipation, *IBS-C* constipation-predominant irritable bowel syndrome, *FED* fecal evacuation disorder, *y* year, *M* male, *F* female

#### Statement No. 6. Abdominal bloating is common in patients with CC

Voting summary: accepted completely 17 (58.6%), accepted with some reservation 11 (37.9%), accepted with major reservation 1 (3%)

Level of evidence: II-2

Grade of recommendation: B

The sensation of abdominal bloating may be reported by as high as 80% of patients with IBS [5, 21, 28]. However, it is not included among the essential symptoms in the Rome IV diagnostic criteria, though the Asian criteria did include it [29, 30]. Patients with IBS-C show more abdominal bloating and/or distention than those with diarrhea-predominant IBS (IBS-D) [1, 28]. In a multicentric international study on IBS, 239 patients were recruited from eight countries, including India [27]. Bowel symptom scale (BSS) was used to assess the severity of symptoms of IBS: pain/discomfort, bloating, constipation, and diarrhea. Symptom scores on bloating were the highest followed by scores on pain and others. India reported the lowest pain and highest constipation scores along with Mexico compared to other countries. Highest bloating scores were reported from Italy followed by India. There was a strong positive correlation between bloating and constipation across all countries and this correlation reached significance for all countries except Italy and China [27]. In another large cross-sectional survey conducted among the adult population in China, 948 out of 16,078 (6%) were diagnosed to have FC by the Rome II criteria. Abdominal bloating was significantly associated with FC and more commonly present among FC patients as compared to IBS-C [31]. Though there is scanty data on frequency of abdominal bloating among patients with FC rather than IBS-C in India, considering the fact that these two conditions are quite overlapping during follow up [32], and have significant pathophysiological similarity [22], the extrapolation of data of IBS-C to FC may not be entirely inappropriate. However, studies from India on frequency of abdominal bloating among patients with FC as compared to IBS-C are needed.

#### Statement No. 7. Detailed clinical evaluation including history and digital rectal examination helps in identifying fecal evacuation disorder

Voting summary: accepted completely 18 (62.1%), accepted with some reservation 8 (27.6%), accepted with major reservation 2 (6.9%)

Level of evidence: II-1

Grade of recommendation: B

Fecal evacuation disorder (FED) can often be suspected and identified from a detailed clinical evaluation including digital rectal examination (DRE) [33]. In one study evaluating the frequency of FED among 249 constipated patients, 86 (34%) were diagnosed as FED by at least any two of the following investigations: abnormal balloon expulsion test, high-resolution anorectal manometry (HRAM), and defecography [22]. Prolonged straining and feeling of incomplete evacuation were the clinical parameters that were significantly associated with the diagnosis of FED [22]. In another study by Shah et al. of 128 patients with CC, 40 (31.2%) were diagnosed as FED based on HRAM findings [25]. Straining at stools, incomplete evacuation, the sensation of anorectal obstruction, and digital evacuation were more likely to be present in patients with FED than without [25]. The importance of a DRE in the diagnosis of FED was emphasized in one of the studies from Korea evaluating 207 patients with FED. DRE showed high sensitivity (93%) and positive predictive value (91%) in detecting FED compared with HRAM [34]. In one Indian study, the sensitivity, specificity, and positive and negative predictive values of DRE in the detection of FED were 69.7%, 81.5%, 82.1%, and 68.75%, respectively among 60 patients with CC [33]. More details about FED will be provided in discussion under statement 22.

#### Statement No. 8. Fulfilling IBS criteria does not exclude fecal evacuation disorder and slow transit constipation

Voting summary: accepted completely 20 (68.9%), accepted with some reservation 6 (20.7%), accepted with major reservation 1 (3%), rejected with reservation 1 (3%)

Level of evidence: II-2

Grade of recommendation: B

Though criteria for the diagnosis of IBS have been clearly defined by the Rome Foundation and Asian consensus [29, 30], FED and slow transit constipation (STC) cannot be ruled out by these criteria alone [22, 25]. This has been emphasized in the multidimensional clinical profile (MDCP) proposed by Rome IV, in which FED and STC are considered to be physiological modifiers of CC. The frequency of FED and STC among patients with IBS has been evaluated by two Indian studies. In one study, the Rome III criteria for the diagnosis of IBS were equally fulfilled by patients with and without FED (74/83 [89%] vs. 117/144 [81.2%]; *p* = ns) [22]. In the other study, the frequency of FED and slow transit constipation was similar between patients fulfilling and not fulfilling IBS criteria (12/25 [48%] vs. 28/74 [38%]; *p* = ns and 4/25 [16%] vs. 11/74 [15%]; *p* = ns, respectively) [25]. Moreover, in a study from Thailand, biofeedback therapy improved IBS symptoms in patients with FED and the response to therapy was similar in patients with and without IBS [35].

#### Statement No. 9. Fecal evacuation disorder is more common among women than among men

Voting summary: accepted completely 15 (51.7%), accepted with some reservation 8 (27.6%), accepted with major reservation 3 (10.3%), rejected with reservation 2 (6.9%), rejected completely 1 (3%)

Level of evidence: II-2

Grade of recommendation: C

Most studies on FED from the West showed a female predominance and there is a common belief that FED is less common among males [36]. But a few hospital-based Indian surveys demonstrated the occurrence of FED among male population as well [22, 25]. The frequency of FED is expected to be higher among women as pelvic floor trauma during labor is considered to be one of the risk factors for FED. In the study of 249 patients with CC, females more often had manometric and defecographic abnormalities compared to males [22]. Intra-rectal and anal pressures during attempted defecation and squeeze pressures were significantly lower among females [22]. In another Indian study, stool frequency of healthy subjects reduced with age starting from 35 years, particularly among female [14]; authors hypothesized that this might be related to the development of pelvic floor dysfunction due to obstetric trauma [14]. In a large study performed among the female general population of Turkey, 4002 were interviewed and about 27% of them reported symptoms of obstructed defecation. Increasing age, vaginal delivery, and higher parity were associated with increased risk of defecatory symptoms related to pelvic floor dysfunction [37]. In a recently reported Indian study on 236 patients with CC, of whom 45 had FED, straining, digital evacuation, and hard stools were more common in females with FED [38].

### Pathophysiology

#### Statement No. 10. Lifestyle factors, some systemic illnesses, several drugs, and physiological abnormalities such as slow colon transit and FED contribute to CC

Voting summary: accepted completely 23 (79.3%), accepted with some reservation 4 (13.8%), accepted with major reservation 1 (3%)

Level of evidence: II-2

Grade of recommendation: B

In epidemiological surveys, lifestyle factors reported to contribute to CC [1] include insufficient dietary fiber and fluid intake, irregular and inadequate time in the toilet, sedentary life, prolonged bed rest, systemic illnesses, and chronic consumption of drugs causing CC [39, 40]. Socio-demographic and lifestyle factors associated with constipation may differ in different regions [41, 42]. A coastal eastern Indian study evaluating defecation frequency and predominant stool forms among 1200 apparently healthy subjects found that female gender, age > 35 years, non-vegetarianism, and sedentary lifestyle were associated with reduced defecation frequency [14]. A community study from northern India found constipation to be more common among females, non-working people, non-vegetarians, and those with lesser fluid and green leafy vegetables/fruits/cereals intake, and poor physical activity [7]. A recent multicenter study from India [15] showed lifestyle factors associated with CC included physical inactivity, posture during defecation, smoking, intake of tea/coffee/alcohol, and animal protein intake. Common co-morbid diseases were hypertension, diabetes mellitus, and dyspepsia. Associated drug intake included antihypertensive and antidiabetic medications, antidepressants, and lipid-lowering drugs. Talley [43] reported a list of drugs that carry a significant risk of constipation; this included antidepressants, antipsychotics, anticonvulsants, antispasmodics, antihistamines, opioid analgesics, diuretics, iron and calcium supplements, and aluminum antacids.

However, in patients with CC in tertiary care practice, slow colonic transit and FED often contribute to CC in addition to lifestyle factors [1]. A study from a tertiary care center in West Bengal reported that 61.5% patients with CC had associated systemic co-morbidities such as diabetes mellitus (17.6%), hypothyroidism (10.5%), organic brain disease (19.8%), or combination of these diseases (13.6%) [16]. Moreover, 37.7% of patients in this study were found to be regularly taking drugs known to cause constipation.

Physiological abnormalities (slow colonic transit, FED, or combination) have been reported in patients with CC from India. In a study from western India [25], of 99 patients with primary constipation, 74 had functional constipation (FC) and 25 had IBS-C as per Rome III criteria. Pathophysiologic sub-types of primary constipation were normal transit constipation (NTC, *n* = 46), slow transit constipation (STC, *n* = 13), dyssynergic defecation (DD, *n* = 38), and DD plus STC (*n* = 2). Thus, 40% patients with primary constipation had FED and these patients were more likely to have a history of finger evacuation, straining, incomplete evacuation, and sensation of anorectal obstruction as compared to those having no DD. FC and IBS-C were clinically and pathophysiologically similar except for abdominal pain. In another study from northern India [22], of a total of 249 consecutive patients with CC (Rome III), 86 (34%) had FED (abnormality in greater than or equal to two tests: balloon expulsion test, anorectal manometry, and defecography). Prolonged straining, incomplete evacuation, and squeeze anal sphincter pressure were significant predictors of FED on multivariate analysis. Manometry and defecography abnormalities were more common among the female FED patients. This is quite anticipated as obstetric trauma is one of the major factors causing FED among the females.

#### Statement No. 11. Obstetric trauma may contribute to the pelvic floor and anorectal abnormalities contributing to CC

Voting summary: accepted completely 19 (65.5%), accepted with some reservation 7 (24.1%), accepted with major reservation 1 (3%), rejected with reservation 1 (3%)

Level of evidence: II-2

Grade of recommendation: B

Defecation and continence require functional integration of the pelvic floor musculature. Damage to pelvic connective tissue, nerves, and muscles during childbirth contributes to the pathogenesis of pelvic floor dysfunction (PFD) [44]. PFD in this setting may manifest as severe constipation, obstructed defecation, rectocele, hemorrhoids, rectal prolapse, or incontinence [45]. Risk factors for PFD in females include older age, higher parity, and vaginal mode of delivery. [46, 47]. Data from India on this issue are, however, limited.

A study on a female general population of Turkey [37] reported a 67.5% prevalence of PFD of at least one major type. The prevalence of constipation and obstructed defecations were 33.2% and 26.8%, respectively. Age, vaginal delivery, and higher parity were found to be the risk factors associated with the development of PFD. A recent study on Lebanese women visiting clinics in a University Medical Center in Beirut [48] reported a 34.6% prevalence of obstructed defecation. Pelvic Floor Bother Questionnaire (PFBQ) scores were found to be significantly higher in those who had ≥ 3 vaginal deliveries. Two studies from South Korea [49, 50] reported a higher frequency of dyssynergic defecation in female constipated patients with prior vaginal delivery as compared to those without.

In a coastal eastern Indian study among 1200 healthy volunteers, the authors found that stool frequency reduced with age, more so in female than male, and such reduction started at the middle of the fourth decade; the authors suggested that this could be related to pelvic floor trauma due to childbirth. This issue requires further study [14]. A recent study from northern India [22] evaluated the frequency, spectrum, and factors associated with FED among patients with CC. This study found that female patients with FED more frequently had (a) abnormal defecography and (b) abnormalities on anorectal manometry. This issue requires further study [14].

#### Statement No. 12. Indian toilet is more physiological than a western toilet for defecation

Voting summary: accepted completely 14 (48.3%), accepted with some reservation 10 (34.5%), accepted with major reservation 3 (10.3%), rejected completely 1 (3%)

Level of evidence: II-2

Grade of recommendation: B

Defecatory postures differ according to culture; squatting and sitting are the most common worldwide [1]. Conventional Indian and Japanese toilets require squatting posture, but more people are gradually switching to western style toilets in urban areas in India. Western toilets need a sitting posture. Squatting is more physiological, ideal, and relaxed posture for defecation as it offers several advantages over “sitting”: (a) leads to better relaxation of puborectalis and hence widening of recto-anal angle; (b) faster, easier, and more complete evacuation; and (c) prevents excessive straining thereby protecting pelvic nerves from becoming stretched and damaged [51, 52]. However, there is limited published literature supporting the advantages of squatting posture. In a study from Israel [53] comparing three postures during defecation (squatting, sitting on standard height toilet seat, and sitting on low height toilet seat) showed that both the time needed for sensation of satisfactory bowel emptying and the degree of subjectively assessed straining were much lower in the squatting position as compared to both the sitting postures. In a recent study from Japan [54] comparing three postures during defecation (squatting, sitting, and sitting with the hip flexed at 60° by placement of the feet on a height-adjustable step) showed that basal abdominal pressure before defecation was at lowest and recto-anal angle on defecation was at widest with squatting as compared to both the sitting postures.

#### Statement No. 13. Bristol stool form correlates with colon transit

Voting summary: accepted completely 19 (65.5%), accepted with some reservation 7 (24.1%), accepted with major reservation 1 (3%), rejected with reservation 2 (6.9%)

Level of evidence: II-2

Grade of recommendation: B

Stool form is often used as a clinical surrogate for colon transit in constipated patients. Bristol stool form scale (BSFS) has been in use in clinical practice for more than two decades [55]. It has proved acceptable both to subjects in epidemiologic surveys and to patients attending gastroenterology clinics. Reasonable correlations have been observed between BSFS and whole-gut transit time [55–57]. The utility of BSFS has been duly endorsed by the Rome Foundation [58].

The validity of BSFS was confirmed in a study from Mayo Clinic, USA [12], which showed that total as well as segmental colonic transit were significantly slower in persons with harder stools (BSFS scores 1–3) than those with looser stools (BSFS scores 5–7). A recent multicenter study from the USA [59] showed a moderate correlation of stool form score with whole-gut and colonic transit times (CTT) in patients with constipation and found that Bristol stool form value < 3 predicted delayed whole-gut and colonic transit with a sensitivity and specificity of more than 80%. A recent multicenter study from Thailand [18] showed that average 5-day BSFS was independently associated with delayed CTT and also that optimal average 5-day BSFS of ≤ 3 provided 68.0% sensitivity, 69.7% specificity, and 69.4% accuracy for predicting delayed CTT. Unfortunately, there is no study on this issue from India.

#### Statement No. 14. Specific pathophysiological abnormalities at the molecular level (myopathic, neuropathic, others) have been shown in a subset of patients with CC

Voting summary: accepted completely 11 (37.9%), accepted with some reservation 14 (48.3%), accepted with major reservation 4 (13.8%), rejected completely 1 (3%)

Level of evidence: II-3

Grade of recommendation: C

A few recent studies suggested that a subset of patients with CC may have some organic basis [60]. Demonstration of histological abnormalities in patients with CC is essentially limited to resected colon specimens [61]. Hence, these results cannot be generalized to patients with CC as colonic resection may be needed only in a small subset of patients with severe CC. Moreover, published literature is limited with most series being small and primarily pertain to STC [62]. Elucidation of histopathological and ultrastructural abnormalities require special techniques like immune-histochemistry (IHC), immunofluorescence, and transmission electron microscopy (TEM). The spectrum of histopathological abnormalities include hypoganglionosis, inflammatory neuronopathy, degenerative leiomyopathy, loss of interstitial cells of Cajal (ICC) or glial cells, decreased acetylcholinesterase activity, mast cell infiltration, and neuroendocrine cell abnormalities [63–66]. There are a few studies published from different parts of the World demonstrating specific pathophysiological abnormalities at the molecular level in patients with CC (Table 4) [62, 63, 67–76] including a few Asian studies [77–79].

**Table 4:** Studies from outside India that reported molecular level pathophysiological abnormalities in patients with chronic constipation

Author, year	Number of subjects	Findings
Park et al. 1995	Idiopathic CC: 14, non-CC controls: 17	Higher number of PGP-9.5 immunoreactive nerve fibers in the muscularis propria [67]
Sjolund et al. 1997	STC: 18	Colonic specimens showed an increased peptide YY and 5-HT containing cells and also increased content of VIP, galanin, substance P, and NPY [68]
Faussone-Pellegrini et al. 1999	STC: 7, non-CC controls: 5	A lower total neuron density and VIP-immunoreactive neurons at the two enteric plexuses reduced NOS-immunoreactive neurons at the myenteric plexus but more NOS-immunoreactive neurons at the submucous plexus [69]
Knowles et al. 2001	STC: 36, controls: 80	Increased frequency of smooth muscle inclusion bodies suggestive of myopathy [63]
Wedel et al. 2002	STC: 11, non-CC controls: 13	Reduced number of ICC, myenteric plexus hypoganglionosis [62]
Lee et al. 2005	STC: 10, non-CC controls: 10	Decreased densities of ICC and PGP 9.5 reactive neuronal structures [70]
Bassotti and Villanacci 2006	STC: 26, non-CC controls: 10	Reduced density of enteric ganglia cells, glial cells, and interstitial cells of Cajal but more apoptotic enteric neurons [71]
Wedel et al. 2006	STC: 13, controls: 12	Myenteric hypoganglionosis, deficiency of ICC, and reduced immunoreactivity to smooth muscle markers [72]
Wang et al. 2008	STC: 15, non-CC controls: 45	Reduced number of ICC and enteric neurofilaments in muscularis propria [73]
Bassotti et al. 2011	STC: 29, non-CC controls: 20	Higher number of mast cells in all colonic segments [66]
Bassotti et al. 2012	FED: 11, non-CC controls: 20	Higher number of mast cells in all colonic segments [74]
Bassotti et al. 2012	FED: 17, non-CC controls: 10	Fewer glial cells in the enteric plexus; and reduced estrogen receptors β in the glial cells [75]
Chan et al. 2013	STC: 61	Reduction in smoothelin immunoreactivity in the muscularis propria [76]

*CC* chronic constipation, *STC* slow transit constipation, *FED* fecal evacuation disorder, *PGP* protein gene product, *5-HT* 5-hydroxytrptamine, *VIP* vasoactive intestinal peptide, *NPY* neuropeptide Y, *NOS* nitric oxide synthase, *ICC* interstitial cell of Cajal

A Korean study [77] evaluating 14 patients with severe idiopathic CC undergoing subtotal colectomy with 10 non-constipated controls revealed myenteric ganglion cells (MGC) and interstitial cells of Cajal (ICC) to be reduced in patients as compared to controls. A study from China [78] evaluated histopathology of 12 patients with STC and 8 age-matched normal controls. ICC was identified with a monoclonal antibody to c-kit by an indirect immunofluorescence method. Patients with STC showed a reduced number of ICC in all four regions (myenteric plexus, submucosal border, circular and longitudinal muscle layers). A study from Japan [79] evaluated rectal biopsy specimens from patients with CC and age-matched normal controls for a number of neuroendocrine cells (NEC) within mucosal crypts using immunohistochemistry with antibodies against chromogranin-A (Ch-A) and serotonin (5-HT). The authors found a number of NEC to be significantly higher in patients with CC compared to controls.

A recent study from Delhi [80] evaluated histopathology of resected colonic specimens from patients with chronic intestinal pseudo-obstruction. Desmosis was seen in all 8 subjects (100%), while intestinal neuronal dysplasia (IND), mesenchymopathy, lymphocytic myenteric ganglionitis, and leiomyopathy were noted in 4, 2, 1, and 1 patients, respectively. One patient with IND also had visceral myopathy.

#### Statement No. 15. Excessive methane production slows gut transit and is associated with CC

Voting summary: accepted completely 15 (51.7%), accepted with some reservation 9 (31.0%), accepted with major reservation 3 (10.3%), rejected completely 1 (3%)

Level of evidence: II-2

Grade of recommendation: B

About 30% to 62% of healthy humans harbor methane-producing bacteria in their gut [81]. In vitro and in vivo studies indicate that methane inhibits GI motility and hence its level may inversely correlate with stool form and frequency [82, 83]. Moreover, the area under the curve (AUC) for breath methane has been shown to correlate with severity of constipation [82]. Furthermore, treatment with antibiotics aimed at gut methanogens has been shown to improve intestinal transit and constipation. A recent meta-analysis [84] found a significant association between methane on a breath test and constipation (odd’s ratio = 3.51) and also an association between methane and delayed transit. There is limited data from India and the rest of Asia on the role of methane in CC.

A recent experimental study from Korea [85] found that infusion of methane significantly decreased peristaltic velocity and increased contraction amplitude of guinea pig ileum. Another study from Korea [86] found breath methane positivity to be significantly more frequent in patients with slow transit constipation than those with normal transit constipation and healthy controls (58.8%, 13.3%, and 12.2%, respectively). The left and total CTT were significantly higher in breath methane positive than negative patients.

A recent Indian study [87] found a higher copy number of *Methanobrevibacter smithii* in fecal samples of patients with IBS (particularly IBS-C) compared to healthy controls. The copy number negatively correlated with the stool frequency and was higher among methane producers than non-producers. The AUC for breath methane correlated with the *M. smithii* copy number among methane producers. A randomized controlled trial from this same center showed that reduction of breath methane using rifaximin shortens CTT and improves constipation [88].

#### Statement No. 16. Psychological issues are common in patients with CC

Voting summary: accepted completely 18 (62.0%), accepted with some reservation 11 (38%)

Level of evidence: II-2

Grade of recommendation: B

Psychological disorders have been reported to be associated with various functional gastrointestinal disorders (FGID’s) including CC and IBS [89]. Many patients with CC have evidence of current or previous psycho-affective disorder [90]. Anxiety, depression, and insomnia are the more commonly reported disorders. Psychological co-morbidities further impair quality of life in patients with CC [91]. Patients with dyssynergic defecation and normal transit constipation or IBS-C are more likely to report psychological distress as compared to STC [92–94]. There is limited published literature from Asia in general and India in particular on frequency and spectrum of psychological co-morbidities in patients with CC.

A study from China [95] reported the presence of anxiety, depression, and sleep disorders in 41.5%, 38.3%, and 43.8%, respectively. These co-morbidities were more common in severely constipated patients than those with mild and moderate constipation; psychological co-morbidities also resulted in more hospital visits. A recent study from Hong Kong [96] showed significantly higher anxiety and depression scores in patients with FC as compared to healthy controls. Anxiety and depression scores were higher in female patients, whereas male patients tended to use more coping strategies, and a number of coping strategies used correlated inversely with anxiety scores.

The landmark pan-Indian study [5] reported a 60% prevalence of anxiety or depression while 39% subjects reported disturbed sleep. A study from New Delhi reported a 79.9% prevalence of ≥ 1 psychological co-morbidity in patients with IBS [97]. The psychiatric co-morbidities observed in this study included anxiety, depression, panic syndrome, and somatoform disorders. Moreover, patients with severe disease had higher psychiatric co-morbidity. A recent study that evaluated frequency and risk factors of FGIDs in a rural Indian population found depression, anxiety, and disturbed sleep to be more common in patients with IBS and dyspepsia as compared to those without FGID [6].

#### Statement No. 17. Solitary rectal ulcer syndrome is associated with FED

Voting summary: accepted completely 21 (72.4%), accepted with some reservation 5 (17.2%), accepted with major reservation 1 (3%), rejected completely 1 (3%)

Level of evidence: II-1

Grade of recommendation: B

Solitary rectal ulcer syndrome (SRUS) is a chronic benign rectal disorder affecting all age groups and presents with rectal bleeding, mucorrhea, tenesmus, constipation, and feeling of incomplete evacuation [98]. Etiopathogenesis of SRUS remains unclear. Mucosal hypoperfusion/ischemia was proposed to be the causative factor in the past [99]. A few recent studies reported the role of FED in the pathogenesis of SRUS [100, 101]. There is limited data from India on the frequency of FED in patients with SRUS.

A case-control study from Lucknow, India [102] showed that (a) 90% of patients with SRUS had CC (as per ROME III criteria), (b) patients with SRUS more often had FED as compared to healthy controls (as documented by balloon expulsion test [BET] and impaired anal relaxation), (c) more than half of patients with SRUS had abnormal defecography, (d) more than 40% of patients had functional defecation disorders according to Rome III criteria, and (e) those with abnormal BET had thicker internal anal sphincter (on endoscopic ultrasound examination) than those without. Another recent case-control study from Varanasi, India [103] showed a higher frequency of FED in patients with SRUS as compared to healthy controls as documented by the presence of abnormal BET and impaired anal relaxation (53% vs. 20%; and 44% vs. 15%, respectively).

### Investigations and treatment

#### Statement No. 18. Alarm feature which may necessitate invasive investigations like colonoscopy includes age > 45 years, visible or occult GI bleed, family history of colon cancer, unintended weight loss, abdominal mass, and fever

Voting summary: accepted completely 24 (82.8%), accepted with some reservation 5 (17.2%)

Level of evidence: II-2

Grade of recommendation: A

**Fig. 1:**
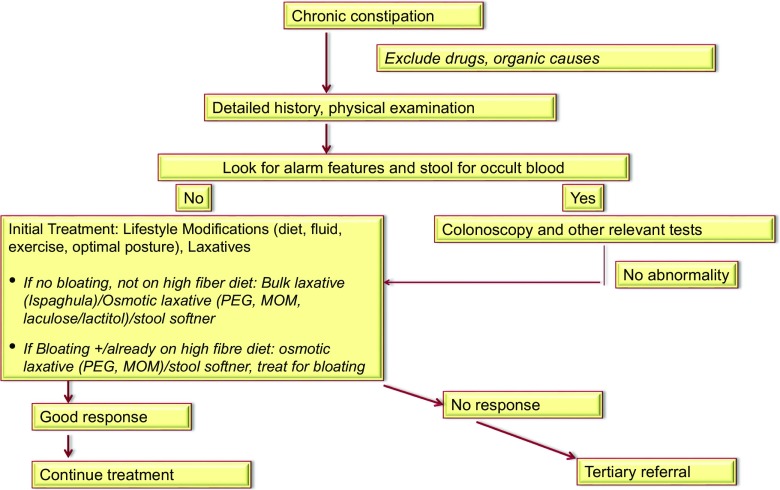
Algorithm for diagnosis and treatment of chronic constipation in adults. *PEG* polyethylene glycol, *MOM* milk of magnesia

The alarm features help to screen patients for possible organic pathologies such as colon cancer (Fig. 1). Several studies, particularly from the West, demonstrated that some of these alarm features may be reasonably sensitive though not specific to suggest the presence of organic causes of CC. However, no study from India evaluated this issue. Moreover, the cut-off age may depend on population prevalence and risk of colon cancer. However, in spite of lack of data from India, screening for these features and, if present, appropriate investigations to exclude organic disease such as colon cancer is advisable. Fecal occult blood test (FOBT) needs special mention in this context as it is an  easily available cheap test to rule out the possibility of colorectal cancer. Most of the colorectal cancer screening guidelines including the Asian guidelines recommend it for screening [104]. FOBT can be immunochemical (fecal immunochemical test, FIT) or guaic based. The FIT is a preferred test as it does not need any dietary restrictions. One-time FIT has been reported to have a sensitivity of 79% (95% confidence interval [CI], 69 to 86) and a specificity of 94% (95% CI, 92 to 95) for detection of colorectal cancer [105].

#### Statement No. 19. Initial treatment of CC should include lifestyle modification and osmotic laxatives

Voting summary: accepted completely 22 (75.8%), accepted with some reservation 6 (20.7%), accepted with major reservation 1 (3%)

Level of evidence: I-1

Grade of recommendation: A

Various lifestyle factors, though not solely responsible, may play some role in patients with CC. Moreover, as these are simple, safe, healthy, and easy to follow practices, these may be recommended by physicians even though the scientific data to prove their efficacy are limited. These measures include adequate dietary fiber intake, drinking plenty of water, regular exercise, and maintaining a healthy sleep pattern. Posture during defecation may have implications in patients with CC. Sakakibara et al. showed that defecating in squatting posture requires less time and effort and is more physiological [54]. Some patients with mild constipation may be treated even by these measures alone. In a Dutch study [106], it was shown that at least 2 L of water per day increased the efficacy of dietary fiber in CC. In another case report, colonic transit was found to increase with exercise [107].

In patients with CC, laxatives are the first line of pharmacotherapy (Fig. 1), which has been listed in the recent review [1]. Osmotic laxatives like lactulose and ispaghula contain non-absorbable molecules which increase the water content in the stool thus softening its consistency and increasing its volume. Stimulant laxatives like bisacodyl, sodium picosulfate, and senna increase the fluid and electrolyte secretion in the lumen and also increase the colonic peristalsis. Although, no head to head trial exists comparing any two types of laxatives but osmotic laxatives are preferred first line treatment by most physicians. In an Indian study [108], it was found that 20 and 30 g of ispaghula was equally effective and more efficacious than 10 g per day dose. In the same study, although stool weight and overall symptom score improved, but whole-gut transit time remained unchanged. However, excess dietary fiber (which are insoluble) and ispaghula (a soluble fiber, the husk of the seed of *Plantago ovata* plant that grows in Indian subcontinent) may increase bloating, though the later is less than the former.

#### Statement No. 20. Fiber supplement should be avoided if the patient is already on high fiber diet and/or abdominal bloating is a prominent symptom

Voting summary: accepted completely 19 (65.5%), accepted with some reservation 7 (24.1%), accepted with major reservation 2 (6.9%), rejected with reservation 1 (3%)

Level of evidence: II-2

Grade of recommendation: B

Fibers are recommended as the first-line treatment by many experts for the treatment of CC, particularly if dietary fiber intake is low and bloating is not a major symptom [109]. The common source of fiber supplementation is ispaghula husk, which is the husk of seeds of *Plantago ovata*, which primarily grows in the Indian subcontinent. This provides soluble fiber in contrast to dietary fibers, which are often insoluble. Insoluble fibers cause more gas and bloating than the soluble fibers. Two studies from northern India reported on the daily fiber intake in patients with IBS. In a study by Malhotra et al. [110], daily crude fiber intake in patients with IBS was found to be low as compared to healthy controls. Diet of IBS patients in this study was found to be low on vegetables and fruits. However, this study included patients with both IBS-C and IBS-D and the authors have not separately analyzed the dietary fiber intake in these two sub-groups. In another study by Singh et al. [111], dietary fiber (soluble plus insoluble) was found to be equal among IBS patients and healthy controls. However, on sub-analysis, crude fiber intake was found to be more in IBS. The total fiber intake in both the groups was found to be around 51 g per day, which is more than the recommended dose. In a randomized trial [112] comparing psyllium with soluble/insoluble fiber for CC, it was shown that bloating and flatulence was more common with insoluble fibers. Though a systematic review evaluating 14 studies [113] found that there was no significant impact of fiber on bloating, many studies included in the meta-analysis did not report on the adverse effect of fiber. One of the studies included in the same meta-analysis showed that 18% of psyllium-treated patients reported abdominal pain compared with 0% of placebo group; however, the pooled symptom scores of digestive system side effects (abdominal pain, flatulence, borborygmi, and bloating) with rye bread was higher than low fiber toast.

#### Statement No. 21. Patients refractory to initial treatment should be investigated for pathophysiological factors like slow colon transit and FED

Voting summary: accepted completely 22 (75.8%), accepted with some reservation 6 (20.7%), accepted with major reservation 1 (3%)

Level of evidence: II-1

Grade of recommendation: A

**Fig. 2:**
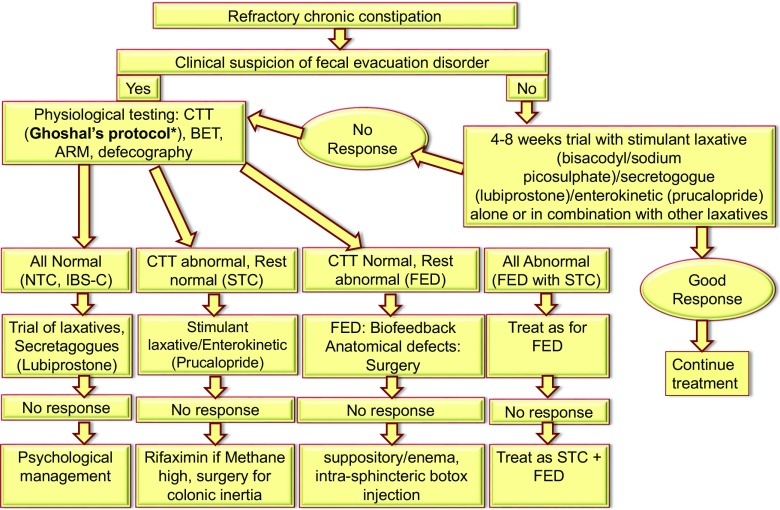
Algorithm for diagnosis and treatment of refractory chronic constipation in adults. *CTT* colon transit time, *BET* balloon expulsion test, *ARM* anorectal manometry, *NTC* normal transit constipation, *IBS-C* constipation-predominant irritable bowel syndrome, *STC* slow transit constipation, *FED* fecal evacuation disorder *Ghoshal UC, et al. Natl Med J India. 2007;20:225–9.

Though most patients presenting to primary care centers may have mild constipation and lifestyle and dietary factors contributing to their symptoms, a refractory condition in tertiary facilities needs to be investigated for the complex pathophysiological issues (Fig. 2), which are not uncommon [22, 25]. In a study from Western India [25], of 128 adult patients with CC, 15% and 40% had STC and FED, respectively. As primary treatment of FED is biofeedback, diagnosis of this condition is important for treatment of this condition. In another study from northern India one-third of patients with CC were found to be having FED [22]. More studies are needed from India on this issue.

#### Statement No. 22. A through rectal examination evaluating resting and squeeze pressure and relaxation during attempted defecation is useful to screen for FED

Voting summary: accepted completely 20 (69.0%), accepted with some reservation 8 (27.6%), accepted with major reservation 1 (3%)

Level of evidence: II-1

Grade of recommendation: A

**Fig. 3:**
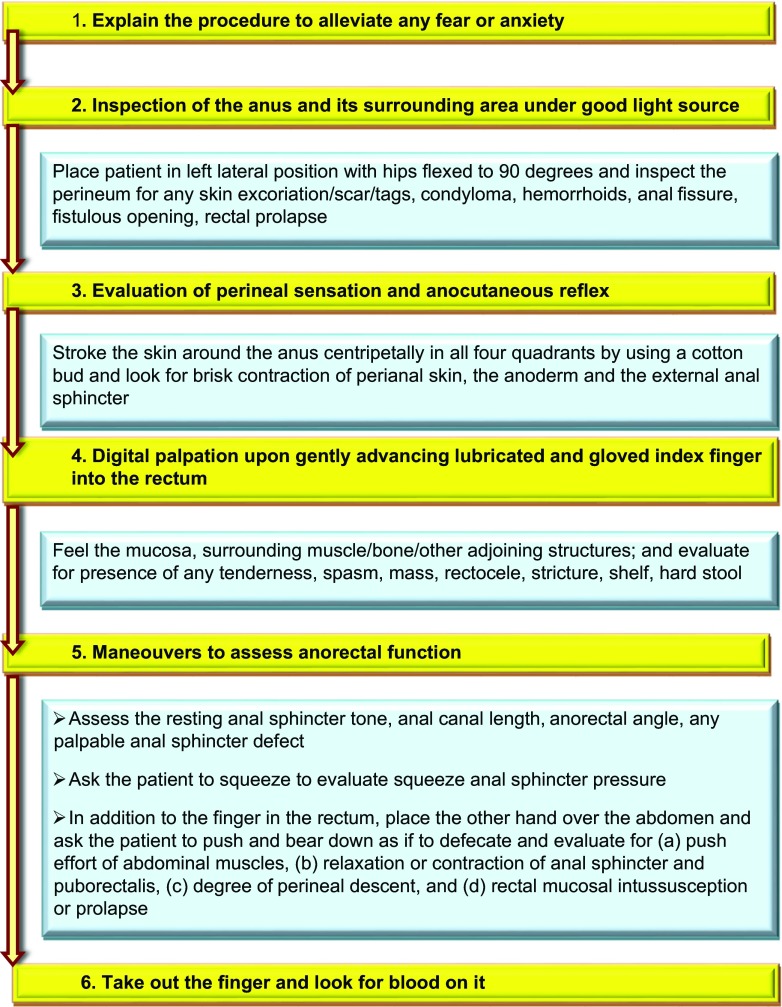
Steps of performing digital rectal examination

Among patients with CC, FED is not uncommon. It is important to recognize this condition as laxative therapy alone is often unsatisfactory among these patients. As anorectal manometry is not widely available, there is a need to know the common clinical parameters indicating the presence of FED in patients with CC. Recently, a few Indian studies addressed this issue. In a recent study, one-third of 249 patients with CC had FED. In this study, prolonged straining (> 30 min), incomplete evacuation, high resting, and squeeze sphincter pressures were associated with FED. In another Indian study, patients with FED more often reported having a digital evacuation, straining, incomplete evacuation, hard stools, and feeling of anorectal obstruction; this study also reported higher resting anal sphincter pressure among patients with FED. Resting and squeeze sphincter pressure can be grossly assessed by digital rectal examination (DRE). In a study from Korea [34], of 309 constipated patients, 207 (77.2%) were diagnosed with dyssynergia using manometry. The sensitivity, specificity, and positive predictive value (PPV) of DRE in the diagnosis of dyssynergia were 93.2%, 58.7%, and 91.0%, respectively, and a moderate agreement was seen between the two modalities (*κ*-coefficient = 0.542, *p* < 0.001). In another study, 73% of patients with FED could be diagnosed by DRE [114]. In a recent Indian study on 60 patients with CC, sensitivity, specificity, PPV, and negative PV of DRE in the detection of FED was 69.7%, 81.5%, 82.1%, and 68.75%, respectively [33]. It is important to recapitulate how DRE is performed as a multicentric study showed that many doctors are not well-versed in performing DRE [115]. Figure 3 shows the steps of performing DRE.

#### Statement No. 23. Western protocol for colonic transit study may not hold well in India

Voting summary: accepted completely 23 (79.3%), accepted with some reservation 4 (13.8%), accepted with major reservation 2 (6.9%)

Level of evidence: II-2

Grade of recommendation: A

The protocol for assessment of colon transit time (CTT) developed in the Western countries may not hold well in India, where the transit is much faster [116]. In quite a few studies, when a Western protocol of administering 20 radio-opaque markers each at 0, 24, and 48 h, and abdominal radiograph at 72 h was used, most of the markers were found expelled even in patients with CC [5]. Hence, the protocol for the CTT was modified. In a study validating a new protocol of performing CTT, 20 radio-opaque markers filled in 2 capsules were administered at 0, 12, and 24 h, and then abdominal radiographs obtained at 36 and 60 h was found useful (**Ghoshal's protocol**). Retention of ≥ 30 radio-opaque markers at 36 h (sensitivity 90%, specificity 82%) and ≥ 14 markers at 60 h (sensitivity 95%, specificity 100%) was quite accurate to detect slow colon transit and FED as causes for CC [116]. Two other Indian centers reported similar results using this modified protocol [117, 11].

#### Statement No. 24. Multiple test positivity including balloon expulsion test, anorectal manometry, and defecography has better accuracy than a single test for diagnosis of FED

Voting summary: accepted completely 22 (75.9%), accepted with some reservation 7 (24.1%)

Level of evidence: II-2

Grade of recommendation: B

Use of a single test, for example, anorectal manometry may over-diagnose FED; in a study from Mayo Clinic, USA, anorectal manometry revealed that a proportion of healthy volunteers were diagnosed having dyssynergic defecation [118]. This might be related to the fact that defecation, which subjects believe should be in utmost privacy, may be difficult in the laboratory environment. This limitation, however, gets obviated in an appropriate clinical setting in patients with CC and positive results in multiple tests (anorectal manometry, BET, CTT, and defecography). Moreover, considerable overlap of colonic transit abnormalities, anatomical abnormalities, and pelvic floor dysfunction occurs in CC. The overlap between slow transit and FED, both of which requires therapeutic attention, is not uncommon. In a recent paper, abnormal results in two out of three studies were diagnostic for FED in 34% patients as compared to single test positivity rate of 61%, 23%, and 56% by defecography, ARM, and BET alone, respectively [22]. BET is a simple bedside test to rule out FED. It is safe, cheap, and gives instantaneous results. It may be especially helpful when rectal manometry is not available. It may be done while lying in left lateral or sitting postures, both of which are comparable [119]. A balloon, tied at end of a thin catheter, is placed inside the rectum and subsequently is filled with 50 mL water; the patient is asked to expel it while lying in left lateral position; if unsuccessful, serially higher weight is added to the other end of the hanging catheter. Inability to expel or need of > 250 g added weight is suggestive of FED. The sensitivity of BET alone for the diagnosis of FED is about 56% [22]. Endoscopic ultrasonography of anal sphincter using the radial endosonoscope at a high frequency (12 Mhz) can be used to study the anatomy of the anal sphincter. In patients with SRU with FED, anal sphincter has been found to be thicker [102].

Though a systematic study is not currently available evaluating sensitivity, specificity, and negative and positive predictive values of various combinations of the anorectal function tests to diagnose FED, futility of a single test such as anorectal manometry to differentiate between patients with CC and healthy volunteers has been shown [118]. Moreover, in Indian study on refractory CC, of 249 patients, though 23%, 56%, and 61% patients had abnormal manometry, non-expulsion of balloon, and abnormal defecography, respectively, 34% had at least two abnormal tests, most of whom respond to biofeedback treatment [22, 120]. More studies are needed on this issue.

#### Statement No. 25. The top-down approach may be preferred in selected patients with CC in the open healthcare system of India, particularly in the tertiary care environment

Voting summary: accepted completely 3 (10.3%), accepted with some reservation 14 (48.3%), accepted with major reservation 9 (31.0%), rejected with reservation 2 (6.9%), rejected completely 1 (3%).

Level of evidence: III

Grade of recommendation: C

Management of CC mainly focuses on symptom relief. Most patients coming to tertiary level healthcare setting have already received most of the conventional treatment like lifestyle and dietary modifications and common laxatives. Most of these patients, therefore, are unlikely to improve and be satisfied with a step-up approach recommended currently as per current guidelines. In fact, a multinational study, patients with CC were found to be most dissatisfied with treatment among patients with FGIDs [121]. Besides thorough investigations, they may improve and be more satisfied with a top-down approach. This approach involves treating the primary pathophysiology first like biofeedback for dyssynergic defecation, colokinetic for slow transit constipation. A randomized controlled trial on 368 patients from 27 centers in the UK showed that bisacodyl, a stimulant laxative not commonly used as first-line therapy of CC as per conventional step-up approach, resulted in 5.2 ± 0.3 spontaneous bowel movement per week compared to 1.9 ± 0.3 on placebo during 1-month follow up [122]*.* The drug was well-tolerated as well. Though only 58.6% experts accepted this statement, we still wish to keep this statement to motivate researchers, particularly from the Asian countries, to undertake more research in this subject as it is very important issue in clinical practice and there is scarcity of data on this issue. However, instead of proceeding to top-down, another practical option may include step-up approach from whatever level the patients was treated in the past.

#### Statement No. 26. Drugs stimulating colonic motility should be preferred for management of slow transit constipation

Voting summary: accepted completely 21 (72.4%), accepted with some reservation 6 (20.7%), accepted with major reservation 2 (6.9%)

Level of evidence: I

Grade of recommendation: A

STC should be treated by colokinetic agents as they target the primary pathophysiological abnormality. Prucalopride, a 5HT_4_ agonist, increases colonic motility. In a European study [123], prucalopride was found to hasten colonic transit (42.8 h in patients on 2 mg prucalopride vs. 54.8 h on placebo). In a randomized controlled trial [124], the proportion of patients with three or more weekly complete spontaneous bowel movements (CSBM) was 30.9% among those receiving 2 mg prucalopride and 28.4% of those receiving 4 mg of prucalopride, as compared to 12.0% receiving placebo [124]. Stimulant laxatives like senna and bisacodyl also increase the colonic transit. In an old Indian study, 69% of patients with advanced cancer and opioid-induced constipation, which is usually slow transit in nature, responded to Sofsena [125].

#### Statement No. 27. Biofeedback should be the initial treatment for FED

Voting summary: accepted completely 17 (58.6%), accepted with some reservation 8 (27.6%), accepted with major reservation 2 (6.9%), rejected with reservation 1 (3%), rejected completely 1 (3%)

Level of evidence: I

Grade of recommendation: A

In a study from Mumbai, 14 (70%) of 20 patients completing more than four sessions of biofeedback had a significant improvement in symptoms and CSBM [25]. Another study from Lucknow, India showed that 62% patients had an improvement in symptoms and anorectal physiological parameters after biofeedback therapy at 1-month follow up [120].

#### Statement No. 28. Surgery should be reserved for patients with refractory CC with specific functional and or structural abnormalities

Voting summary: accepted completely 21 (72.4%), accepted with some reservation 6 (20.7%), accepted with major reservation 2 (6.9%)

Level of evidence: II-2

Grade of recommendation: B

The literature on surgery for CC is scanty from India. In a study from a tertiary level teaching hospital in northern India, 34 patients with refractory CC were treated surgically over a 6-year period [126]. This study showed that spontaneous bowel movement increased following surgery. However, it is important to note that these patients were highly selected to include patients with severe STC, large rectocele, and adult Hirschsprung’s disease. Hence, the results of this study must not be extrapolated to unselected patients with refractory CC.

#### Statement No. 29. Psychological evaluation must be performed before surgical treatment

Voting summary: accepted completely 17 (58.6%), accepted with some reservation 11 (37.9%), accepted with major reservation 1 (3%),

Level of evidence: II-2

Grade of recommendation: B

Psychological evaluation and proper counseling regarding the post-surgical outcome must be performed before surgical treatment. In a study from the UK where 44 women with refractory constipation underwent colectomy, 10 were found to have a psychiatric illness which was partly contributing to their primary illness [127]. Although many publications are there from Asia on surgery for CC, very few of them talk about psychological evaluation and its impact on surgical outcome. The psychological evaluation is particularly important to exclude patients having such co-morbidity from surgical management with potential for non-response.
